# An Annual Plant Growth Proxy in the Mojave Desert Using MODIS-EVI Data

**DOI:** 10.3390/s8127792

**Published:** 2008-12-03

**Authors:** Cynthia S.A. Wallace, Kathryn A. Thomas

**Affiliations:** 1 U.S. Geological Survey, Western Geographic Science Center, 520 North Park, Tucson, Arizona 85719, USA; 2 U.S. Geological Survey, Southwest Biological Science Center, 106 Environmental and Natural Resource Building, University of Arizona, Tucson, Arizona 85721-0120, USA; E-mail: Kathryn_A_Thomas@usgs.gov

**Keywords:** Annual vegetation, MODIS EVI, Mojave Desert

## Abstract

In the arid Mojave Desert, the phenological response of vegetation is largely dependent upon the timing and amount of rainfall, and maps of annual plant cover at any one point in time can vary widely. Our study developed relative annual plant growth models as proxies for annual plant cover using metrics that captured phenological variability in Moderate-Resolution Imaging Spectroradiometer (MODIS) Enhanced Vegetation Index (EVI) satellite images. We used landscape phenologies revealed in MODIS data together with ecological knowledge of annual plant seasonality to develop a suite of metrics to describe annual growth on a yearly basis. Each of these metrics was applied to temporally-composited MODIS-EVI images to develop a relative model of annual growth. Each model was evaluated by testing how well it predicted field estimates of annual cover collected during 2003 and 2005 at the Mojave National Preserve. The best performing metric was the spring difference metric, which compared the average of three spring MODIS-EVI composites of a given year to that of 2002, a year of record drought. The spring difference metric showed correlations with annual plant cover of R^2^ = 0.61 for 2005 and R^2^ = 0.47 for 2003. Although the correlation is moderate, we consider it supportive given the characteristics of the field data, which were collected for a different study in a localized area and are not ideal for calibration to MODIS pixels. A proxy for annual growth potential was developed from the spring difference metric of 2005 for use as an environmental data layer in desert tortoise habitat modeling. The application of the spring difference metric to other imagery years presents potential for other applications such as fuels, invasive species, and dust-emission monitoring in the Mojave Desert.

## 1. Introduction

Qualitative modeling of species habitat is best done with environmental data inputs that are relevant to the biogeography of the species of concern [1]. However, the availability of digital data for these ecologically relevant factors is often limiting. Time and financial resources can constrain the effort to collect, analyze, and compile new data sources for digital maps. In addition, an ecologically relevant environmental factor may be difficult to capture directly. An alternative to omitting potentially important data sources in a modeling effort is to use existing data resources to develop best estimates or proxies for the environmental variable of concern.

**Figure 1.**
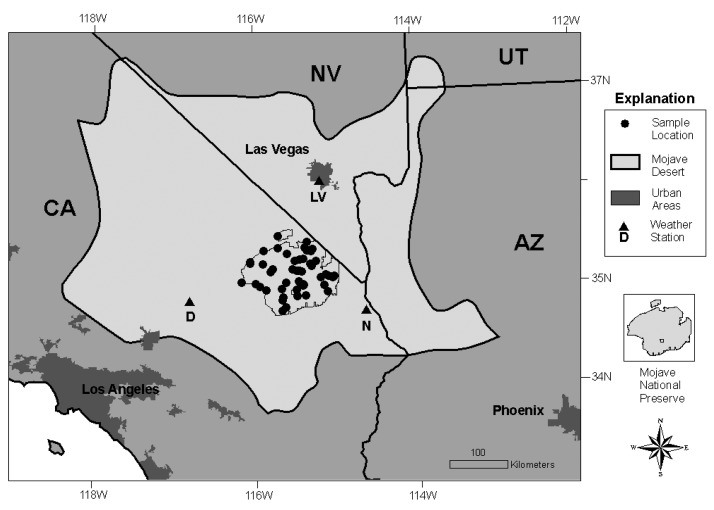
Map of the Mojave Desert ecosystem study area and the field sample locations. Weather stations shown are Las Vegas (LV), Daggett (D) and Needles (N).

The Mojave Desert in the southwestern United States (U.S.) (Figure 1) supports habitat for the federally listed Mojave Desert tortoise (*Gopherus agassizii*) [2]. The Department of Interior asked the U.S. Geological Survey to develop a potential habitat map for the desert tortoise using existing data inputs such as tortoise surveys, digital maps of environmental factors, and remotely sensed images. The assembled U.S. Geological Survey team included desert tortoise biologists who identified environmental factors known to be important to desert tortoise biology as suggested model inputs. Included among those factors was the potential of an area to support annual plants over time, a capacity we refer to as annual growth potential.

In the arid environment of the Mojave Desert, annual plant growth, as measured by annual cover, is largely dependent upon the timing and amount of rainfall [3, 4]. Annual plants may be prolific in one year and not the next, and they may be prolific in one area of the Mojave and simultaneously absent in another suitable area within the Mojave. When annual plants are present, they are most obvious in the interspaces between sparsely spaced shrubs, but they can also occur under the shrubs in so-called “shrub islands” [5]. The spatial, temporal, and compositional complexity of annual plants in the Mojave Desert has made mapping of annual plant cover at any one point in time technically complex and, for the purposes of characterizing tortoise habitat, ecologically uninformative.

In this paper, we present our approach to developing a proxy of annual plant growth for use as an environmental layer in desert tortoise habitat modeling. We developed provisional models using multiple Moderate-Resolution Imaging Spectroradiometer (MODIS) Enhanced Vegetation Index (EVI) satellite images and metrics designed to capture the plant phenology revealed in these data. Our proxy integrates apparent annual plant response over a number of years and expresses the abiotic potential of the landscape to support annual plant growth under sufficient precipitation.

## 2. Background

### 2.1. The Mojave Desert

The Mojave Desert encompasses about 115,000 km^2^ of arid landscape in southern Nevada, western Arizona, southwestern Utah, and southeastern California (Figure 1). Nearly two million people live in this desert, concentrated in large cities such as Las Vegas and Lancaster. However, forty million people in southern California and central Arizona are within a day's drive. Much of the Mojave Desert is under federal management, including four national park units, six major military training bases, and considerable acreage of BLM land.

**Figure 2.**
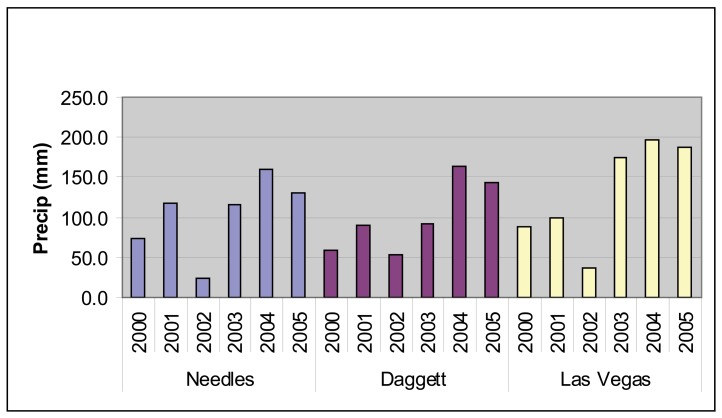
Annual precipitation recorded at three geographically isolated weather stations from 2000 to 2005 within the Mojave Desert. Both the temporal and spatial variability of precipitation are illustrated. The weather stations represent landforms and elevations where desert tortoise habitat is expected within the Mojave with Needles at 288 m, Dagget 588 m, and Las Vegas 658 m. The year 2002 was particularly dry while 2004 and 2005 had high annual precipitation. (Data source: Western Regional Climate Center, http://www.wrcc.dri.edu/)

**Figure 3.**
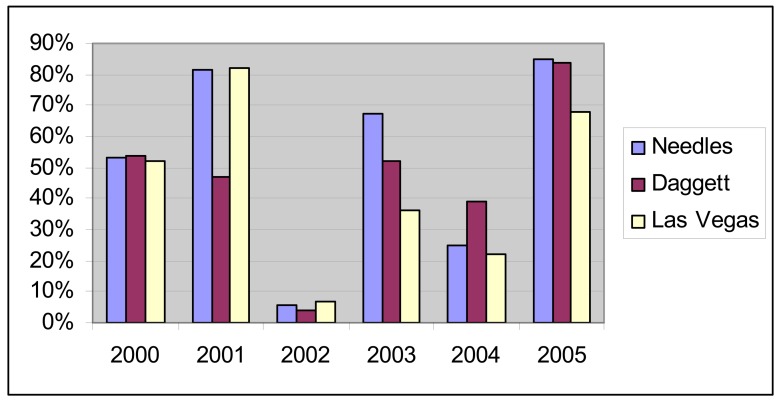
Percentage of yearly precipitation that occurred during January, February, and March of 2000 through 2005 at three weather stations in the Mojave Desert. Little precipitation fell in 2002 during the critical establishment season for annual plants. In contrast, 2005 had a large percentage of the annual precipitation falling during the critical establishment season for annual plants at all three stations. (Data source: Western Regional Climate Center, http://www.wrcc.dri.edu/)

The Mojave has cold winters and very hot summers [6, 7]. Precipitation varies with elevation and as shown in Figure 2, precipitation totals are both variable from year to year and geographically variable within a single year. Throughout the Mojave, 2002 was a particularly dry year while 2004 and 2005 had strong precipitation (Figure 2). However, the spring of 2004 was uncharacteristically dry and most of the precipitation occurred later in the year (Figure 3).

The Mojave Desert supports plant communities comprised of annual grasses and forbs, biennial and perennial shrubs, perennial grasses, and succulent plants such as agaves, cacti, and yuccas. The percentage of perennial plant cover is low and estimates based on remotely sensed images have indicated 28% of the Mojave has less than 10% perennial plant cover and 67% of the Mojave has less than 20% plant cover (Wallace, unpublished data). Annual plants consist of ephemeral native and non-native plants that appear seasonally when moisture is sufficient. Green-up of the annual plants is generally strongest in the early to mid spring [7, 8, 9], after which the annual grasses and forbs generally dry up. When summer rains are adequate, there may be an additional green-up following summer rains, most often seen in the higher elevations of the eastern Mojave in response to summer storms coming from the southeast. Perennial plants likewise respond to precipitation. Many shrubs are drought deciduous and drop part or all of their leaves when rainfall is lower. Shrubs and small trees will green-up in the late spring when rainfall is adequate. Perennial grasses are generally a small component of each plant community and, as distinct plant communities, are found in only localized areas of the Mojave. The perennial grasses green-up later in the spring and summer compared to the annual plants. Succulents also generally occur as scattered components in plant communities except for a few areas in the Mojave. The succulents generally maintain leaves year round, when present, and do not display a distinct green-up period.

### 2.2. Satellite imagery

Many studies use satellite-derived vegetation indices, such as the Normalized Difference Vegetation Index (NDVI) [14], coupled with field data to estimate total biomass, cover, or net primary productivity [10-13]. Multitemporal imagery, which allows the incorporation of phenological differences among image dates, is used increasingly to eliminate the problem of distinction between soil and vegetation signal in arid and semi-arid environments [14-16]. A number of studies derived greenness indices for arid grasslands from multitemporal images using MODIS, Advanced Very High Resolution Radiometer (AVHRR) or Landsat Thematic Mapper (TM) imagery [17, 18, 19]. Peterson [20] focused on a single plant species in Nevada and used two dates of Landsat data in a model trained with field-measured data to map the cover of *Bromus tectorum* (cheatgrass), a nonnative grass. Wallace *et al.* [17] applied stepwise linear regressions between field measurements of perennial cover and MODIS-EVI data to model regional perennial vegetation cover in the Mojave Desert. These studies typically train their vegetation models with field data they collect, using the field data as the dependent variable and deriving the relationship to the image values used as independent variables in regression analysis.

MODIS- EVI images provide regional coverage, capture the dynamics of vegetation distributions across the landscape, are calibrated, and are freely [21] available from the USGS Global Visualization Viewer website (http://glovis.usgs.gov). These attributes help quantify even the small amounts of vegetation found in sparse deserts, because differences between the images are dominated by differences in vegetation phenology, with soils and topographic effects remaining relatively constant across adjacent images, if not across years.

MODIS data are collected daily at 250-meter resolution in the red (620-670 nm) and near-infrared bands (841-876 nm) and at 500-meter resolution for the blue band (459-479 nm) [21]. The EVI product is a spectral measure of the amount of photosynthetically-active vegetation on the ground, calculated using the red, near-infrared (NIR), and blue bands, as follows:

(1) $$\text{EVI}=G\frac{\rho_{\text{NIR}}-\rho_{\text{RED}}}{\rho_{\text{NIR}}+C_{1}\times \rho_{\text{RED}}-C_{2}\times \rho_{\text{BLUE}}+L}$$

Where *ρ* is the atmospherically corrected or partially atmospherically-corrected (Rayleigh and ozone absorption) surface reflectance, *L* is the canopy background adjustment that addresses nonlinear, differential NIR and red radiant transfer through a canopy, and *C_1_*, *C_2_* are the coefficients of the aerosol resistance term, which uses the blue band to correct for aerosol influences in the red band. The coefficients adopted in the *EVI* algorithm are, *L* = 1, *C_1_* = 6, *C_2_* = 7.5, and *G* (gain factor) = 2.5 [16]. The *EVI* equation optimizes the vegetation signal, de-couples the canopy background signal, and reduces atmospheric influences to allow for precise inter-comparisons of spatial and temporal variations in terrestrial photosynthetic activity [16]. Daily MODIS-EVI data are combined into single 16-day composites using an improved Constrained View Maximum Value Composite (CV-MVC) scheme that reduces sun-target-sensor angular variations. Each year comprises twenty-three 16-day composites.

## 3. Data

### 3.1. MODIS-EVI images

MODIS-EVI satellite data provide a regional view of the Mojave ecosystem captured over 16-day intervals. Landscapes contain a mix of perennial and annual vegetation types that green up at rates and times characteristic for their distinct species, and these phenologies are captured by MODIS data [17, 18, 20]. We used 115 MODIS-EVI images for 2001-2005.

EVI values are delivered as 16-bit numbers scaled to a valid range from -2,000 to 10,000, in which non-land surfaces (such as water or snow) typically assume negative values and land surfaces typically assume positive values [16]. As landscapes become more densely vegetated, the calculated EVI approaches 10,000. To reduce data volumes, we rescaled the 16-bit data to 8-bit as follows:

(2) $${\text{EVI}}_{8-\text{bit}}=[({\text{EVI}}_{16-\text{bit}}÷10000_{\left(\text{ScaleFactor}\right)})+1]*100$$

This rescaling shifts the valid EVI _16-bit_ values to the range of 80 to 200, with a calculated EVI _16-bit_ = 0 shifted to EVI _8-bit_ = 100. We inspected each image for non-data (non-valid) values (flagged with EVI _16-bit_ = -3000) and observed only scattered pixels in water bodies. These non-data pixels are represented by an EVI _8-bit_ value of 70; they do not represent land surfaces and were not involved in model development.

### 3.2. Evaluation data

We used annual cover estimates made at observation sites in the Mojave National Preserve to evaluate the performance of the relative annual growth models. These data were collected for a previous study. The locations were initially selected using April 1992 Landsat Thematic Mapper (TM) imagery, which represented a wet month in a wet year for the Mojave Desert, to select locales with strong vegetation signature and nearby locales with weak vegetation signature. These sites excluded bedrock mountainous erosional highlands. Geographic coordinates of the sites were field recorded, and each site center was marked for relocation in the field.

In the spring of 2003 and 2005, field observers estimated the total perennial plant cover, total annual cover, non-native annual forb cover, and non-native annual grass cover at the selected sites. The visual estimates were for an area represented by a circle extending 50 meters from the site center (approximately .75 ha). The field crew, consisting of two experienced botanists, approximated the observation boundaries by pacing with a range finder. The observers calibrated their ocular estimates to a measured area on the ground and then made independent estimates of cover. Any differences in cover estimates were reconciled among the observers. In 2003, the sites were visited four times during the spring and the maximum annual cover among those visits was used. In 2005, 37 of the sites were visited once in late April. Not all 50 sites were revisited due to time and resource constraints. The 2003 field effort originated for a separate project and the 2005 revisits were done to capture the abundant annuals present that year for comparison.

## 4. Model development

### 4.1. Approach

In contrast to previous studies, in this project we created a number of provisional models of relative annual growth using satellite data alone, with field data used to evaluate rather than to calibrate the models. We identified groupings of the MODIS-EVI images that captured different growing season characteristics of annual plants in the Mojave and then developed twelve metrics that incorporated these groupings to create provisional annual growth models. Model performance was evaluated with the existing data collected in the Mojave in 2003 and repeat measures of the same sites in 2005.

### 4.2. Phenological groupings and reference points

**Figure 4.**
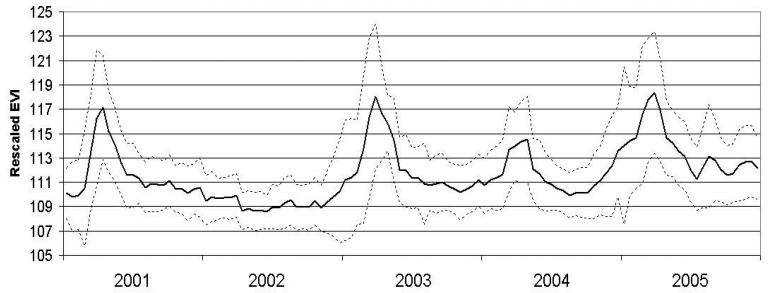
Profile of the average MODIS-EVI for all 50 field-sites at each MODIS composite time period during 2001 - 2005 (23 per year). Solid line shows Mean of 50 sites, with dashed lines defining +/- 1 Standard Deviation.

We inspected the MODIS-EVI values at all 50 field sites averaged from 2001 through 2005 to determine the relationship of the values with the expected phenological response of desert vegetation (Figure 4).

We assembled the MODIS-EVI images into the following groupings based on the phenology profile above and input from desert ecologists, and roughly labeled them according to seasons:

1. Spring: Composites 4, 5, and 6 with composite start dates of February 18, March 6, and March 22, respectively. The Mojave Desert typically exhibits a flush of annual greenness in the early springtime and many areas host their most abundant annual cover at that time. The MODIS-EVI profile for that period is typically highest.
2. Early Summer: Composites 10, 11, 12, and 13 with composite start dates of May 25, June 10, June 26, and July 12, respectively. Annual plants in the Mojave Desert typically dry up as rainfall diminishes and the temperature rises. The MODIS-EVI profile for this period is typically lowest.
3. Winter: Composites 20-23 of the previous year (composite start dates November 1, 17 and December 3, 19) and composites 1-8 of the current year (composite start dates January 1, 17, February 2, 18, March 6, 22, and April 7, 23). Annual plants may begin to respond to precipitation as early as November of the previous year and grow through the early spring.
4. Late Summer: Composites 15, 16, and 17 with composite start dates of August 13, 29 and September 14. In some Mojave Desert landscapes, a pulse of annual vegetation may be present in the fall as a response to summer rains.

For the period of this study (2000 to 2005), the MODIS-EVI profile (Figure 4) and field observations (Thomas pers. obs.) showed virtually no annual vegetation in 2002 and high annual vegetation in 2005. The year 2002 was an exceptionally dry year throughout the Mojave Desert and in particular for the critical growing season for annuals (Figures 2 and 3). In comparison, 2005 was a wet year with high precipitation in the critical growing season following a wet year with lower precipitation in the critical growing season (Figures 2 and 3). Based on the contrasts in image values and precipitation patterns between these two years, we identified 2002 as a reference year for very low annual growth and 2005 as a reference year for high annual growth.

Several of the provisional models we tested compare the average image values for a particular season for a year to that same season in 2002. Our rationale was that environmental factors such as sun angle, soils, substrate, and topographic shadowing remain constant for a given time of year, so subtracting the average image values observed in 2002 will compensate for the spectral contributions of these factors. Perennial plants also respond to drought, as evidenced by leaf drop and subdued green-up. However, unlike annual plants, which respond to extreme drought by not germinating, i.e. being nonexistent, the structure of a perennial plant remains and contributes to the satellite signal. Therefore, although differences in greenness of the perennial vegetation due to relative climate conditions are expected, we assume the signal of the annual vegetation is dominant and that the difference between the seasonal image values for a given year and the image values of 2002 will primarily reflect the relative amount of annual growth in a given year.

### 4.3. Annual growth metrics

Inspection of the MODIS-EVI profile (Figure 4) reveals the vegetation “greenness” dynamics of the landscape. There are several ways to capture measures of this profile, including basic statistics, form fitting and Fourier transforms [22-25]. For this study, basic statistics were used to derive metrics based on seasonal groupings of selected MODIS composites and Fourier transforms were used to derive metrics based on the entire MODIS-EVI profile for each individual year. Applying Fourier analysis, we treat the ordered 23 EVI values at each pixel as a vector (or waveform) and extract the additive term (best fit of a flat line to the profile), and the first frequency magnitude and phase (the amplitude and timing of the peak for the best fit of a single sine wave to the profile). These measures capture information on vegetation vigor or density, timing of maximum greenness, and variability of greenness, respectively [22, 23]. Table 1 shows the 12 metrics we derived and tested as possible measures related to annual growth.

### 4.4. Annual growth model development and evaluation

We created 24 provisional models of relative annual growth applying the 12 test metrics to MODIS-EVI images for 2002, 2003, and 2005 and evaluated the models using the field estimates of annual plant cover for 2003 and 2005. For each field site, we extracted the model values for 2005 (37 sites) and 2003 (50 sites) and calculated a linear regression between the field-estimated annual cover (percent) and the model predictions of annual growth. We assumed the provisional model that best predicted the measured annual-cover amounts at the field sites was superior. Clearly, there are potential problems with this assumption because: (1) the annual-cover estimates were collected in the local area of the Mojave National Preserve and are, therefore, not necessarily representative of the entire Mojave Desert and, (2) the field data were collected for a different study and were, therefore, not necessarily representative of 250m MODIS pixels (see [10] and [26] for MODIS sampling strategies). Given these potential problems, we consider a significant fit between the annual-growth model and the field data to be fortuitous and evidence that the metric applied was a reasonable approximation of relative annual growth.

## 5. Results

**Figure 5.**
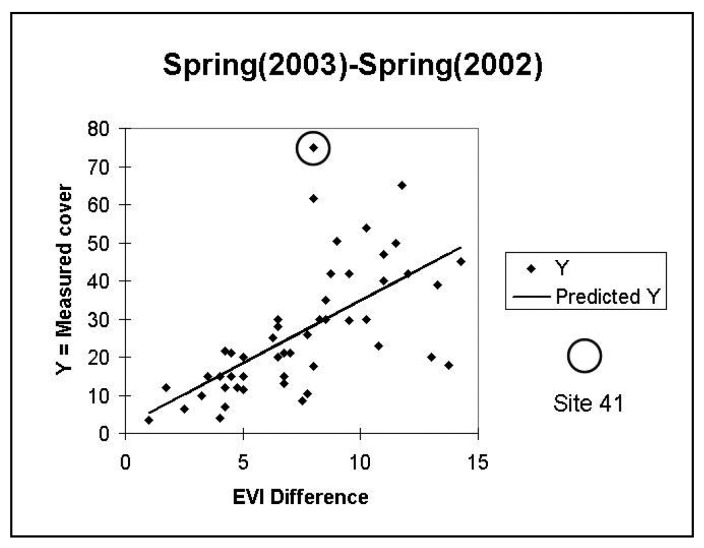
Scatter plot between field-estimated 2003 annual cover and the annual-growth model based on the metric Average Spring (2003)-Average Spring (2002). Site 41 was anomalous because in spring 2003 it had the highest total perennial plus annual coverage of all sites, at 93 %. (Y= Annual Cover, measured in field and predicted).

**Figure 6.**
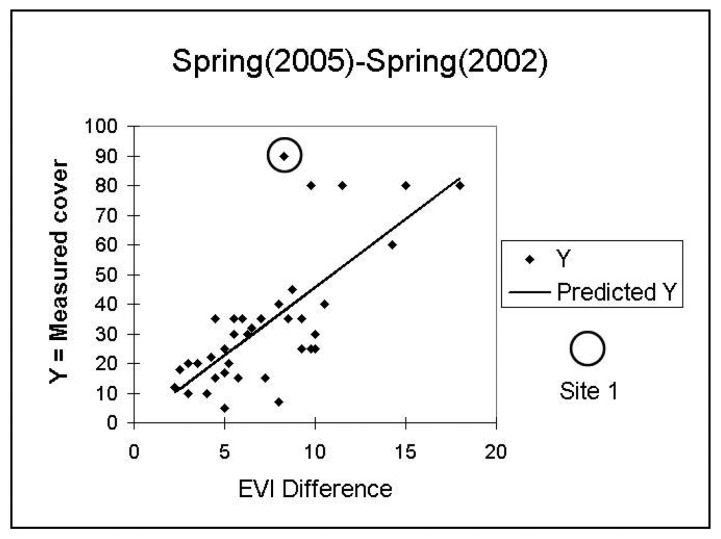
Scatter plot between field-estimated 2005 annual cover and the annual-growth model based on the metric Average Spring (2005)-Average Spring (2002). In spring 2005, site 1 had nearly 90% annual cover and, in contrast to other high-annual-cover sites that were dominated by forbs, it was composed of nearly all non-native *Schismus*. (Y= Annual Cover, measured in field and predicted).

Initial investigations of the relationship of the field-estimated annual cover with model values revealed apparent outliers, site 41 for 2003 and site 1 for 2005 (see Figures 5 and 6). The field notes for these sites reveal that they are anomalous. On 4/24/2003, site 41 had the highest total perennial plus annual coverage of all sites, at 93 %. On 4/21/05, site 1 had nearly 90% annual cover and, in contrast to other high-annual cover-sites that were dominated by forbs, it was composed of nearly all non-native *Schismus*. The vegetation composition of other high cover sites was inspected and did not reveal any distinct compositional differences. We excluded sites 41 and 1 from further analysis.

**Figure 7.**
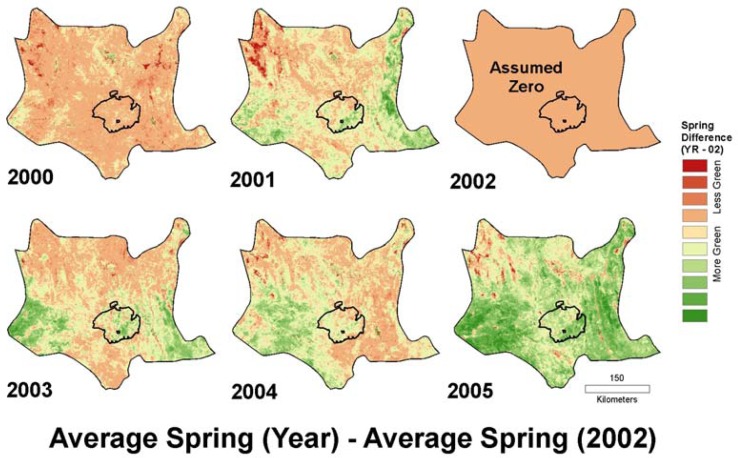
Annual growth models for 2000 to 2005 represented by the best performing metric Average Spring (Year) - Average Spring (2002). The gradual color ramp of pink to green represents low to high annual growth. These are created by assuming the spring annual plant cover for 2002 (an extremely dry year) was essentially zero.

Table 2 summarizes the results of the linear regressions between the suite of provisional relative annual growth models and the field-estimated annual cover data from 2003 and 2005. The highest regression result was R^2^ = 0.61 significant at p < 0.01 between measured 2005 annual cover and the model based on the metric Average Spring (2005)-Average Spring (2002). This metric also gave the highest correlation (R^2^ = 0.47, significant at p < 0.01) for field-estimated 2003 annual cover. The annual growth models represented by this spring difference metric for 2000 to 2005 are shown in Figure 7.

Inspection of additional results (Table 2) revealed several significant correlations, but with low R^2^ in both years for annual growth models based on metrics that that included some part of the spring grouping and models based on the Fourier Magnitude difference. “Late Summer” based models were not correlated, “Early Summer” based models were poorly correlated, and the models based on “Winter” groupings were inconsistent.

Our original purpose for this study was to create a proxy for annual growth potential of the landscape for use in desert tortoise habitat modeling. We ultimately chose to use a rescaled version of the spring difference model calculated for the year 2005. Not only did this model have the highest R^2^, it is ecologically intuitive in that it evaluates the typical green-up period for annuals and captures the high contrast of annual response between an exceptionally wet spring (2005) and exceptionally dry spring (2002).

High elevation landscapes in the northwest Mojave Desert show low annual response for the 2005 spring difference model (Figure 7), which is likely due to the presence of snow persisting at higher elevations later into the spring of 2005 compared to 2002. As an environmental layer for the tortoise, we maintained this contrast, since desert tortoises are not found at these higher elevations.

We rescaled the model values into 8-bit data with a range between 0 and 200 using the following formula:

(3) $$\left\{\frac{\left[\left(\text{Average Spring}\;(2005)\right)-\left(\text{Average Spring}\;(2002)\right)\right]}{\left[\left(\text{Average Spring}\;(2005)+\text{Average Spring}\;(2002)\right)\right]}+1\right\}*100$$

**Figure 8.**
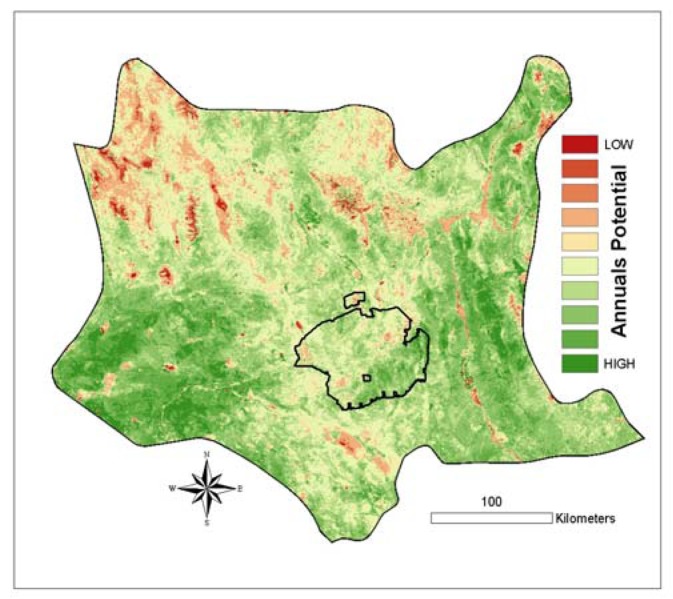
Proxy for annual growth potential in the Mojave Desert based on analysis of MODIS-EVI data. In this image, which is a re-scaled version of the 2005 model shown in Figure 7 (see text), areas with greener values correspond to areas predicted to have high annual growth potential.

This formula is analogous to a Normalized Difference Vegetation Index (NDVI), which extracts a vegetation signal by contrasting the high near-infrared (NIR) reflectance with the low red (RED) reflectance value, calculated as: [(NIR-RED) / (NIR+RED)]. In our case (equation 3), the high value is Spring 2005 greenness and the low value is Spring 2002 greenness. The resulting image (Figure 8) was applied as the proxy in the tortoise modeling effort of the landscape's potential to support annual plants.

## 6. Discussion

We evaluated seasonal groupings of MODIS-EVI images and identified a metric to use as a functional proxy for annual growth potential that correlated with annual plant cover as estimated in a restricted portion of the Mojave Desert. Our study found the strongest correlation between measurements of annual plant cover and MODIS derivatives using the satellite metric defined as the difference between the average spring greenness for 2005, a wet year, and the average spring greenness for 2002, a year of record drought. These two years represent recent extremes in climate conditions, with essentially no annual plant production during the 2002 drought year and abundant annual plant growth during the record rainfall year of 2005. As such, the spring greenness observed in 2002 captures invariant environmental factors, including sun angle, soils, substrate, topographic shadowing and, to some extent, perennial vegetation cover. Our study created provisional models using basic statistics and Fourier Transforms, which capture information on selected portions of the annual greenness curve and on the entire curve, respectively. Other tools exist for developing phenological metrics and could be examined for refinement of this approach. For example, phenological metrics extracted using difference metrics, smoothing filters, and fitting functions calculated using TIMESAT [27] are used to evaluate the effect of pre-fire fuels treatments and to monitor recovery in the Rodeo-Chediski fire perimeter of central Arizona [24].

It is important to remember that this study used the field data only to evaluate the provisional satellite-based models and not for calibration in model development. Also remember these data are from the local area of the Mojave National Preserve and are not necessarily representative of the entire Mojave Desert. Because of these limitations, this model is not a calibrated map of annual vegetation cover, but is instead an environmental layer intended specifically for habitat modeling - a layer that captures a relative index of potential annual plant response. Although the model could be improved with additional field data collected using methods designed for MODIS pixels that represent all landscape types in the Mojave Desert, it is worth noting that this environmental data layer was found to be highly predictive of desert tortoise presence in the tortoise habitat modeling effort [Gass et al., in prep].

While the project had a pragmatic goal and was able to leverage limited resources, the study identified the value of using MODIS imagery from the spring of 2002 as a reference to index relative annual growth in other years. The metric Average Spring (Year) - Average Spring (2002) applied to MODIS-EVI images potentially provides a means to monitor annual growth in any year of consideration. This capacity could be of use in yearly fire-management planning, landscape-level invasive species surveys, and prediction of dust emissions in the Mojave. Since we could only provide a limited test of the efficacy of the spring difference metric, additional field work is needed to fully verify the approach and potentially to calibrate the approach to particular vegetation communities within the Mojave Desert.

Additional analysis of the yearly models created with the spring difference metric could include composites of the models displayed in Figure 7 (e.g., retaining the highest or lowest greenness value at each pixel), measures of variability in annual growth across years (e.g. the range, coefficient of variation, and standard deviation) as well as trends in greenness for two or more years. Since the predictability of forage across years is an important consideration for any species, the landscape variability for annual growth captured by such metrics may also be useful in determining the distribution of desert herbivores.

## 7. Summary

Regional maps that provide information on annual plant cover are critical for informed natural-resource management of habitat because these plants are a seasonally fluctuating food base for many species. Despite the importance of such maps, their creation is hampered by the ephemeral nature of the vegetation they hope to capture and by the remoteness and extent of the areas for which they are needed, as well as by lack of funding to collect and analyze field data. Our study demonstrated that a successful proxy of annual growth potential can be created using MODIS-EVI satellite data alone by examining landscape phenologies revealed in these data combined with expert knowledge of annual plant seasonality. Field data were used to evaluate satellite-based models but were not required for model development or calibration. This study revealed the usefulness of contrasting two years of extremes-in our case comparing 2002 and 2005, a year of record drought with little evidence of annual growth and a year of exceptional spring precipitation with abundant annual growth, respectively. Our final model, found to be predictive of tortoise presence in the habitat modeling effort, is intuitively and ecologically satisfying in that it contrasts the greenness of the two extreme years during the season of expected maximum annual vegetation activity. This type of spatially distributed information is critical to many environmental tasks and is particularly well suited to the development of habitat suitability models for important or endangered species, in our case the desert tortoise.

## Figures and Tables

**Table 1.** Metrics tested to capture annual growth in the Mojave Desert, with supporting rationale.

**Metrics Evaluated for Annual Growth Models**	**Rationale**
Average Spring (year)	This is the average of the spring grouping of MODIS images.
Average Early Summer (year)	This is the average of the early summer grouping of MODIS images.
Maximum Winter (year)	This is the maximum value of the winter grouping of MODIS images. We chose to avoid calculating an average value for this grouping; an average value of these images is likely to be confounded by non-vegetation-related factors such as snow cover.
Average Late Summer (year)	This is the average of the late summer grouping of MODIS images.
Average Spring (year) / Average Early Summer (year)	This ratio is defined to enhance the extreme difference between spring green-up and early summer senescence of annuals.
Maximum Winter (year) -Average Early Summer (year)	This ratio is defined to enhance the extreme difference between full growing season (winter grouping) and early summer senescence of annuals.
Maximum Winter (year) -Maximum Early Summer (year)	This ratio is defined to enhance the extreme difference between full growing season (winter grouping) and early summer senescence of annuals. The maximum summer value used here is just a slight variation of the average early summer value used above.
Average Spring (year) – Average Spring (2002)	The spring green-up of a particular year is compared to the reference year 2002 in which there was virtually no annual response.
Average Late Summer (year) -Average Late Summer (2002)	For areas that experience a pulse of late summer green-up, this difference should preferentially capture the amount of annual vegetation during a given year, with 2002 a reference year representing virtually no annual vegetation signal.
[Average Spring (year) – Average Spring (2002)] + [Average Late Summer (year) – Average Late Summer (2002)]	The spring and late summer green-up of a particular year is compared to the reference year 2002 in which there was virtually no annual response. This is the sum of the two preceding metrics.
Fourier Magnitude (year) -Fourier Magnitude (2002)	The Fourier Magnitude is similar to the spring-difference measure above, with 2002 a reference year representing no annual vegetation signal. The magnitude, however, is based on a sine curve fitted to the entire annual profile, as opposed to an average of a single season.
Fourier Additive (year) - Fourier Additive (2002)	The Fourier Additive difference is also similar to the spring difference measure, with 2002 a reference year representing no annual vegetation signal. The additive term, however, is based on a flat line fitted to the entire annual profile (i.e., an average of the entire annual profile) as opposed to an average of a single season.

**Table 2.** Regression results between values predicted by the yearly relative annual growth models and annual cover field estimated in 2003 and in 2005. Forty-nine points were used for the regression using 2003 data, and thirty-six points were used for the 2005 regressions. Insignificant regression results are shown as ***.

	**Year annual cover data collected**			
	**2003**		**2005**	
**Annual Growth Metric**	**R^2^**	**Sig F**	**R^2^**	**Sig F**
Average Spring (year)	0.46	<0.01	0.54	<0.01
Average Early Summer (year)	0.13	<0.01	0.11	<0.01
Maximum Winter (year)	0.42	<0.01	0.28	<0.01
Average Late Summer (year)	***	***	****	***
Average Spring (year) / Average Early Summer (year)	0.40	<0.01	0.48	<0.01
Average Spring (year) - Average Early Summer (year)	0.40	<0.01	0.49	<0.01
Average Spring (year) - Average Spring (2002)	0.47	<0.01	0.61	<0.01
Maximum Winter (year)-Average Early Summer (year)	0.36	<0.01	0.51	<0.01
Maximum Winter (year)-Maximum Early Summer (year)	0.31	<0.01	0.24	<0.01
Average Late Summer (year) - Average Late Summer (2002)	***	***	***	***
Fourier Magnitude(year) - Fourier Magnitude(2002)	0.46	<0.01	0.39	<0.01
Fourier Additive (year) - Fourier Additive (2002)	0.18	<0.01	0.32	<0.01
[Average Spring (year) - Average Spring (2002)] + [Average Late Summer (year) - Average Late Summer (2002)]	0.33	<0.01	0.45	<0.01
